# Gene finding in the chicken genome

**DOI:** 10.1186/1471-2105-6-131

**Published:** 2005-05-30

**Authors:** Eduardo Eyras, Alexandre Reymond, Robert Castelo, Jacqueline M Bye, Francisco Camara, Paul Flicek, Elizabeth J Huckle, Genis Parra, David D Shteynberg, Carine Wyss, Jane Rogers, Stylianos E Antonarakis, Ewan Birney, Roderic Guigo, Michael R Brent

**Affiliations:** 1 Research Group in Biomedical Informatics, Institut Municipal d'Investigacio Medica/Universitat Pompeu Fabra/Centre de Regulacio Genomica, E08003 Barcelona, Catalonia, Spain; 2 Department of Genetic Medicine and Development, University of Geneva, Medical School and University Hospital of Geneva, CMU, 1, rue Michel Servet, 1211 Geneva, Switzerland; 3 Center for Integrative Genomics, University of Lausanne, 1015 Lausanne, Switzerland; 4 The Wellcome Trust Sanger Institute, Wellcome Trust Genome Campus, Hinxton, Cambridge CB10 1SA, UK; 5 Laboratory for Computational Genomics and Department of Computer Science, Campus Box 1045, Washington University, One Brookings Drive, St Louis, Missouri 63130, USA; 6 EBI, Wellcome Trust Genome Campus, Hinxton, Cambridge CB10 1SD, UK

## Abstract

**Background:**

Despite the continuous production of genome sequence for a number of organisms, reliable, comprehensive, and cost effective gene prediction remains problematic. This is particularly true for genomes for which there is not a large collection of known gene sequences, such as the recently published chicken genome. We used the chicken sequence to test comparative and homology-based gene-finding methods followed by experimental validation as an effective genome annotation method.

**Results:**

We performed experimental evaluation by RT-PCR of three different computational gene finders, Ensembl, SGP2 and TWINSCAN, applied to the chicken genome. A Venn diagram was computed and each component of it was evaluated. The results showed that *de novo *comparative methods can identify up to about 700 chicken genes with no previous evidence of expression, and can correctly extend about 40% of homology-based predictions at the 5' end.

**Conclusions:**

*De novo *comparative gene prediction followed by experimental verification is effective at enhancing the annotation of the newly sequenced genomes provided by standard homology-based methods.

## Background

The draft sequence of the chicken (*Gallus gallus*) genome has been recently obtained and an initial analysis completed [1]. This genome sequence will be extremely valuable for vertebrate development, avian biology, and agriculture. To reap these benefits, however, we must be able to determine with reasonable accuracy the amino acid sequences of the proteins encoded by this genome. We therefore set out to adapt three leading gene prediction systems to the chicken genome sequence and to evaluate their predictions experimentally. Some features of the chicken genome facilitate gene prediction while others hinder it. Generally, gene prediction tends to be more accurate in more compact genomes than in the large mammalian genomes [2-6]. The chicken genome is about 40% the size of the human genome, but about three times the size of the Takifugu genome [7-11]. This translates into a substantial reduction in repeats and pseudogenes. Interspersed repeats cover about 9% of the genome, far less than the 40–50% found in mammals [1]. The small number of processed pseudogenes is beneficial to automated annotation, as this technology often misclassifies pseudogenes as functional. *De novo *prediction methods need full open reading frame (ORF) mRNAs to train their statistical models and homology-based methods rely on these sequences in order to predict a core set of high quality genes. However, an abundant set of chicken full-length cDNAs was not available at the start of this project, as only about 1,800 putatively full-length cDNAs and 340,000 ESTs were deposited in GenBank [12].

We set out to determine how well the computational methods used for annotating protein-coding genes in the mouse and rat genomes [9,10] would perform in this avian context, where transcriptome sequencing was much less advanced and where sequence divergence may be beyond the optimal distance. An assessment of the computational methods was carried out by a large-scale experimental verification by RT-PCR. The gene prediction tools tested were Ensembl, SGP2 and TWINSCAN. Ensembl is a homology-based method, which builds gene-models using species-specific known sequences and proteins from other species aligned to the genome [13]. SGP2 and TWINSCAN are *de novo *comparative gene predictors whose only inputs are the genome to be annotated and a second informant genome [3,4,14-16]. In this case, the informant genome selected for comparison was human. Since Ensembl relies on mapping known genes from chicken and other organisms to the chicken genome, we surmised that its prediction set would contain fewer false positives (predicted genes that are not real) and more accurate gene structures, as well as more false negatives (real genes that are not predicted) than the *de novo *methods. Conversely, SGP2 and TWINSCAN should be effective in detecting *bona fide *genes missed by the Ensembl pipeline. In particular, we were able to verify gene structures simultaneously predicted by both *de novo *methods about 50% of the time. In this respect, *de novo *comparative methods complement homology-based methods, which in general miss genes for which there is no pre-existing evidence of transcription.

To test and compare the performance of these prediction systems under the new conditions presented by the chicken genome, we tested a large number of predicted genes by RT-PCR and direct sequencing. We sampled individually from the predictions for which either one or two of the three methods agreed, and the remainder did not. We thus focused our tests on the most difficult genes to predict and on the differences between the various prediction systems. We present here the largest experimental comparison of multiple gene-prediction programs to date as well as the first to use this kind of differential design. We aimed to evaluate how *de novo *and homology-based gene finding methods perform in a newly sequenced genome for which a small number of gene sequences are known. In particular we have (1) evaluated the three prediction methods, (2) investigated TWINSCAN and SGP2 as possible effective methods to complement the Ensembl prediction pipeline and (3) tested the overall specificity of the Ensembl prediction set.

## Results and discussion

### Experimental evaluation of gene finding in chicken

**Figure 1:**
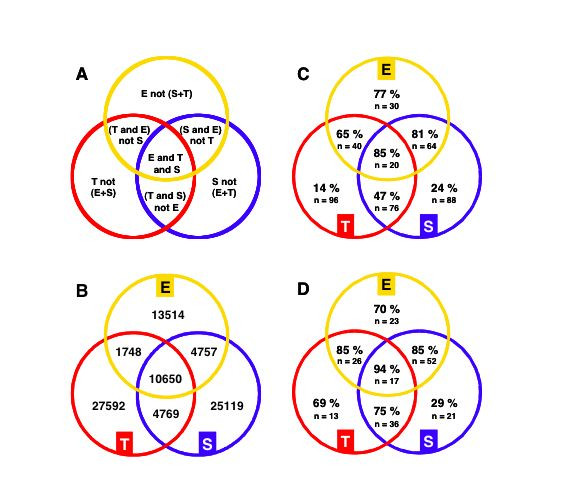
**Venn diagram of the prediction sets. **Venn diagram obtained from the comparison of the three prediction sets: Ensembl (E), SGP2 (S) and TWINSCAN (T). (A) Description of each subset in the Venn diagram. (B) Total number of intron assemblies (IAs) populating each subset. (C) Percentage of experimentally verified IAs for each subset (top) and number of assayed IAs (bottom). (D) Percentage of correctly predicted splice junctions (top) from the experimentally verified IAs (bottom).

**Figure 2:**
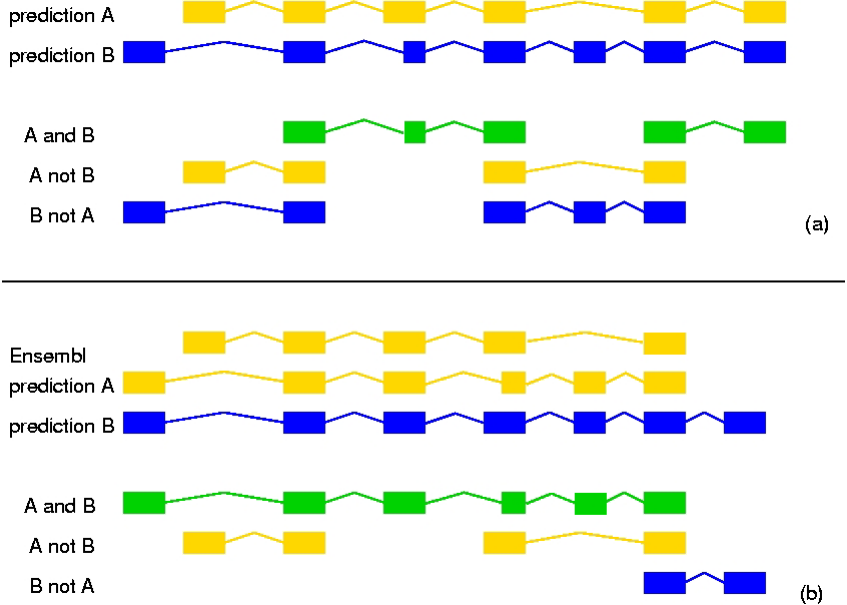
**Comparison of two predictions. **From the comparison of two predictions (a) we obtain three differentiated sets of intron assemblies (IAs): the set of IAs that are identical in both transcripts ('A and B'), and two set of the IAs that are in one prediction but not in the other ('A not B' and 'B not A'). When two sets have the same intron with different outside boundaries for the flanking exons these boundaries are taken from the intersection of the exons. Ensembl predictions (b) have in general more than one transcript per gene (two top yellow tracks). The intersecting intron assemblies (IAs) are therefore defined as the longest non-redundant IAs common between the transcripts from either prediction. For the novel IAs we take the longest non-redundant IAs in one that are not present in the other set.

We aimed to estimate the accuracy of each of the gene prediction sets, which consisted of 29,430 SGP2 (S) and 29,052 TWINSCAN (T) gene predictions, with one transcript per gene, and 17,709 Ensembl (E) genes containing 28,416 transcripts. We classified the predictions according to the Venn diagram defined by the three-way intersections of the sets and their complements (Figure 1A, Table 1). The subsets were populated with intron assemblies (IAs), defined as a list of exons and introns contiguous in a predicted transcript (see Figure 2 and Methods for details), and can be classified into three types: (1) the orphan subsets, containing those elements that are in one set but not in any of the other two, (2) the two-way intersection subsets, containing those elements that are in two sets and not in the third, and (3) the triple intersection, containing those elements that are in all three sets. Subsequently we tested pairs of adjacent exons from each of the subsets.

### Complementing homology-based gene prediction with *de novo *methods

Selected IAs belonging to the three possible two-way intersections were experimentally tested. The results are summarized in Figure 1 and detailed in Table 2. After RT-PCR, gel purification, and direct sequencing, about 50% of the tested transcripts predicted by both SGP2 and TWINSCAN, but not by Ensembl yielded spliced alignments to the gene targeted (Figure 1C). This rate is higher than that reported for human using a combination of homology-based and single-genome predictors [16,17] in spite of the lack of available known gene sequences for the chicken genome. There is a total of 4,769 IAs in the '(S and T) not E' subset, corresponding to a total of 13,470 exons. Projecting these results onto genes and using an average distribution of coding exons per gene from other vertebrates (Human, Mouse and Rat), we estimate that approximately 740 to 840 *bona fide *chicken genes that are not in the currently predicted Ensembl set can be found by the *de novo *comparative methods followed by direct amplification and sequencing.

Considering the set of IAs unique to one prediction set (orphans), 39% of these have one single intron and 80% have 1 or 2 introns. On the other hand, after testing experimentally 96 orphans from TWINSCAN, 88 orphans from SGP2 and 30 orphans from Ensembl, we found that about 77% of the Ensembl orphans are real genes, compared to an average 18% for TWINSCAN and SGP2 (see Figure 1, see Table 2). Thus while Ensembl orphans are more likely to be real genes not predicted by the other methods, orphan *de novo *predictions are more likely to be false positives.

### Extending homology-based gene predictions with *de novo *methods

**Figure 3:**
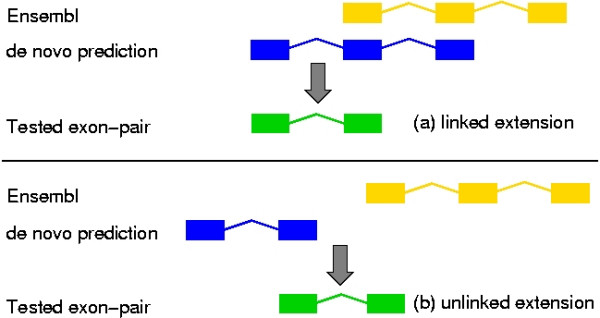
**Ensembl extensions**. Exon extensions to Ensembl predictions can be obtained from exons predicted by TWINSCAN and/or SGP2. These exons can either (a) be part of a transcript with exons in common with the Ensembl transcript (linked) or (b) be part of a close but non-overlapping transcript (unlinked).

As Ensembl predictions often fail to correctly predict one or both ends of a gene [13], we reasoned that *de novo *prediction methods could help in extending the homology-based predicted transcripts. To test this hypothesis, we identified candidate 5' extensions: exons predicted by TWINSCAN, SGP2 or both to the 5' side of Ensembl genes on the same strand. We found that 8,368 (47%) Ensembl genes have such candidate extensions. However, not all these extensions were as likely to correspond to real exons. From this total, 7,630 genes had extensions suggested by *de novo *predictions that overlap the Ensembl gene (linked, see Figure 3A), and 738 (4%) had extra exons from *de novo *predictions that did not overlap the Ensembl gene (unlinked, see Figure 3B). As 99% of Ensembl introns were no longer than 100 kb, we considered only exon extensions that were no further than 100 kb from the 5'-most Ensembl exon. Interestingly, we found that 93% of the linked extensions were to multiexonic Ensembl transcripts, the remainder being extensions to single-exon Ensembl transcripts; however, for unlinked extensions, only 58% were to multiexonic Ensembl transcripts.

We investigated experimentally 60 linked and 29 unlinked extensions by designing one primer in the 5'-most exon of the Ensembl prediction and the second primer within one of the upstream exon suggested by TWINSCAN and/or SGP2 to the 5' side. The RT-PCR results (see Table 3) show that *de novo *methods-suggested linked extensions were correct in about 40% of the cases. This rate dropped to a mere 7% for the unlinked extensions.

Separating the extensions according to whether the extra exon was predicted either by SGP2 or by TWINSCAN showed that both methods had a comparable contribution. From the 60 linked tested extensions, 46 were predicted by SGP2 with 21 (46%) RT-PCR positives, 36 were predicted by TWINSCAN with 15 (42%) positives, and 22 were predicted by both programs with 12 (54%) positives. On the other hand, from the 29 tested unlinked extensions 15 were predicted by SGP2, with 2 positives, and 17 were predicted by TWINSCAN, with 1 positive. Finally, there were 3 cases where both, SGP2 and TWINSCAN, predicted the unlinked extra exon of which one was positive.

### Testing Ensembl specificity

A randomly selected set of Ensembl predictions was assayed to evaluate Ensembl's specificity. This test measured a false positive rate of 4% (see Table S5 of the supplementary material for more details). On the other hand, the tested exon-pairs for the two-way intersection sets that included Ensembl ('(E and S) not T' and '(T and E) not S') had an average false positive rate of 19% and 35%, respectively (see Figure 1). The disparity is greater with the two-way intersection set that excludes Ensembl ('(S and T) not E'), which shows a false positive rate of 53%. One explanation for this difference is the observation that most of the Ensembl predictions have exons predicted by both SGP2 and TWINSCAN. Indeed, 25,222 (89%) of the 28,416 Ensembl transcripts have at least one exon, which is also in the SGP2 and TWINSCAN sets, and 82% of these transcripts have 2 or more exons in common with both *de novo *methods. Thus, Ensembl predictions are most likely to fall within a triple intersection, resulting in an increased rate of true positives. Based on previous experiments [16], we expected the triple intersection to give a yield close to 100% positive rate. We tested 20 cases of triple intersection and found 85% positive rate (see Figure 1C). Moreover, 94% of these positive cases had the exon-intron boundaries correctly predicted (see Figure 1D).

We compared the accuracy of the human-based predictions with the accuracy of a fish-based set of predictions. We predicted genes in chicken with SGP2 and TWINSCAN using Tetraodon nigroviridis as informant genome, and found that they are less accurate than the human-based ones (see Table S7 of the supplementary material). Additionally, we found that 85% and 98% of the Tetraodon-based predictions from TWINSCAN and SGP2, respectively, overlap the corresponding human-based ones. Interestingly, 85% of the TWINSCAN orphans overlap TWINSCAN Tetraodon-based predictions, and 80% of the SGP2 orphans overlap SGP2 Tetraodon-based predictions. On the other hand, 99% of the IAs common to TWINSCAN and SGP2 but not in Ensembl, overlap TWINSCAN or SGP2 Tetraodon-based predictions.

## Conclusions

In this paper we have evaluated how effective purely computational approaches for genome annotation can be, even in the absence of a large collection of previously known genes, by means of the largest attempt so far to experimentally compare several gene finders. After testing the accuracy of Ensembl, SGP2 and TWINSCAN on the chicken genome we have shown that de novo comparative methods followed by experimental verification remain a successful approach in the annotation of newly sequenced genomes from which little is known.

We found that approximately 50% of predictions that were in TWINSCAN and SGP2 but not in Ensembl could be experimentally verified (Figure 1). These experiments demonstrate that *de novo *comparative prediction methods are effective at complementing homology-based methods and confirm that a combination of methods can improve the prediction accuracy [18-22]. Moreover, in spite of the limited gene sequence data available for chicken, the combination of TWINSCAN and SGP2 achieves better accuracy than previous attempts to verify by RT-PCR computational predictions that fall outside a set of annotations [17,23]. On the other hand, looking at the intron assemblies unique to one prediction set, the proportion of positives is largely reduced for predictions not in Ensembl. The predictions unique to one of the *de novo *methods show an abundance of gene models with 2 and 3 exons, which may be artefacts due to genome misassemblies. These results are in contrast with the high success rate (77%) of the predictions unique to Ensembl. This is a reasonable observation considering that the Ensembl prediction pipeline has access to genes that do not follow a 'standard' gene-grammar (e.g., unusual codon usage), but which may nevertheless be represented in the cDNA/protein databases used.

The Ensembl chicken gene set has been found to have a 96% positive rate, whereas the IAs from the two-way intersections that include Ensembl, '(E and S) not T' and '(T and E) not S', and the Ensembl orphans, have a lower positive rate, 81%, 65% and 77%, respectively, which stems from the fact that most exons predicted by Ensembl are also predicted by both SGP2 and TWINSCAN. Additionally, *de novo *comparative methods are useful for extending partial predictions from homology-based methods. Ensembl may generate predictions based on protein fragments or on partial homology from other species, and TWINSCAN and SGP2 predictions can add *bona fide *exons to the Ensembl predictions they overlap with. For the 5' end we show that 40% of the tested cases, where either TWINSCAN or SGP2 predicted at least one additional exon, were verified (Table 3). To our knowledge, this is the first time that experimental evidence is provided for extensions to homology-based models produced by *de novo *methods.

We observed that the subsets containing SGP2 IAs (e.g., '(S and E) not T)') have in general a higher proportion of RT-PCR positives than those containing TWINSCAN IAs (e.g., '(T and E) not S)') (Figure 1C, Table 2). There are two factors that may contribute to this difference. The first is an intrinsic difference between TWINSCAN and SGP2 – SGP2 uses TBLASTX (translated) alignments between human and chicken to reward exons overlapping aligned regions, whereas TWINSCAN uses BLASTN (nucleotide) alignments to influence the scores of exons, splice sites, and translation initiation and termination sites. Human and chicken are sufficiently diverged that translated alignments may be more sensitive, whereas nucleotide alignments fail to cover many known exons. The other factor is incidental to the way TWINSCAN was trained and run to produce the predictions tested. TWINSCAN used 525 chicken RefSeqs to estimate parameters for its probability model. This training set was probably too small to produce optimal parameter values. SGP2, on the other hand, was run with a combination of parameters estimated from the much larger set of known human genes (for its model of chicken DNA sequence) and parameters were hand tuned using the same 525 chicken genes (for its scoring of human-chicken alignments). Although a larger fraction of SGP2 predictions yielded positive experimental results, we found that TWINSCAN tends to be more accurate than SGP2 in the prediction of the intron boundaries (Figure 1D, Table 2). This difference stems from the intron model used by TWINSCAN, as opposed to SGP2, which does not model introns explicitly. TWINSCAN was re-run after completion of the experiments with an improved intron-length model, yielding a prediction set that was substantially smaller and more accurate (see Table S6) than the set tested. In spite these differences, comparing the gene predictions with a set of coding cDNAs released after the completion of these analyses, we found that all three methods have similar sensitivity (79%) (see Methods for details), hence the *de novo *comparative methods cover a fraction of the transcriptome similar to homology-based methods with a minimal initial amount of genome-specific expression data.

The experimentally verified IAs represent a fraction of the actual number of chicken genes that can be eventually found using our methods. If we extrapolate the proportions of experimentally verified IAs (Figure 1C) to all the generated IAs in the Venn diagram (Figure 1B) and using an average distribution of coding exons per gene from other vertebrates (Human, Mouse and Rat), we estimate a range of 14,600 to 17,500 experimentally verifiable chicken genes from our computational predictions. In this paper we only analysed intron assemblies and deliberately left out a number of chicken protein-coding intronless predictions (3,049 from SGP2, 2,727 from TWINSCAN and 1,855 from Ensembl). The triple intersection of these intronless genes contains 148 genes, which are worth investigating and for which techniques different from the ones applied here will be required.

Considering all 2-way IAs (see Table 1), one would need 11,274 RT-PCR reactions to experimentally confirm about 7,232 (64%) genes. This number of experiments compares favourably to large-scale EST projects with the added benefit of having almost no redundancy (only gene fissions and misassemblies will contribute to redundancy). The biggest drawback to EST sequencing is its large redundancy and extensive overlap. The falling cost of primers and the increased flexibility of large-scale molecular biology centers make this approach of computational prediction followed by experimental verification cost effective and scalable [5,6]. As RT-PCR primers can be designed with appropriate linker sites such an approach could also provide a physical resource of clonable fragments. We conclude that *de novo *comparative gene predictions followed by experimental verification is an effective way to carry out the annotation of a newly sequenced genome for which little gene sequence information is known. In particular, as our results show, performing RT-PCR and sequencing for all the predicted novel genes, starting with those predicted by multiple *de novo *methods, should enhance the quality of the annotation in forthcoming eukaryote genome sequencing projects.

## Methods

### Generation of predictions

The initial lack of an abundant set of known chicken gene sequences forced us to adapt the methodology for training of the *de novo *methods and running the Ensembl gene build. The Ensembl prediction pipeline [13] builds gene models from known vertebrate proteins and cDNA sequences, whereas gene models based solely on ESTs are usually kept separated as ESTGenes [24]. For the chicken genome, ESTs were combined with the standard gene build to include additional genes and transcripts. The alignments from approximately 400,000 ESTs and 24,000 full-length cDNAs [12,25] from multiple tissues were conciliated into non-redundant transcript structures [24]. Those predicted models that fell on non-annotated loci and those that contributed with at least two new exons in previously annotated loci were added to the protein-based gene set. Single-exons transcripts produced by the EST/cDNA-based models were rejected, as they could not be distinguished from cloning artefacts without protein evidence. For our analyses we did not include the untranslated regions that Ensembl annotates combining the cDNA/EST and protein evidence [13]. Ensembl also annotates processed pseudogenes [13], which we did not consider either.

TWINSCAN was trained on 525 chicken RefSeq sequences [26] aligned to the chicken genomic sequence. This set is based on a set of 1266 provisional RefSeq mRNAs from GenBank (27 March 2004) March 27 filtered in the following way: The Refseq mRNA sequences were matched with the corresponding mRNAs placed on the Chicken genome assembly at UCSC. Any mRNA not placed was not considered. Additionally, sequences without an ungapped alignment between the entire CDS portion of the Refseq mRNA and the extracted unmasked genomic sequence were removed. Additionally, cases with in-frame stop codons and/or non-canonical splice sites were also removed. TWINSCAN uses nucleotide alignments and has specific models for how the alignments modify the scores of the gene signals. The BLASTN [27] alignments used by TWINSCAN covered 3.8% of the chicken assembly.

SGP2 training followed a hybrid approach. SGP2 was run with human parameters for the coding statistics and splice sites, whereas the score weights that reward the human-chicken homologies and penalize the lack of them were optimised using the same set of 525 Chicken RefSeqs used for the TWINSCAN training. SGP2 was then run on unsegmented chicken chromosomes using the TBLASTX [27] alignments with the human genomic sequence (assembly NCBI34). These alignments, which covered approximately 3% of the Chicken genome, were enriched with 391,610 extra HSPs obtained from the ungapped Exonerate [28] alignments of human proteins from the Ensembl predictions (release NCBI34c), the GeneId prediction set for the same human assembly and the set of vertebrate RefSeq proteins (version of April 2004). The extra alignments covered 43% of the nucleotides in TBLASTX HSPs and 5% of their sequence represented 5,840 non-redundant homology regions that had no overlap with the TBLASTX hits. These extra alignments produced a considerable improvement of the sensitivity and specificity at the gene level with respect to SGP2 predictions using only TBLASTX HSPs when tested against the Ensembl set and the aforementioned 525 RefSeqs. It also achieved a slight improvement of the sensitivity at the exon and nucleotide level.

### Classification of predictions

We classified the predictions according to the Venn diagram defined by the intersections of the three sets: Ensembl (E), TWINSCAN (T) and SGP2 (S). Using the exact identity between transcripts is problematic because the exon-structures from each prediction are in general not identical. Thus we defined the intron assembly (IA) as the element of comparison. An IA is a list of exons and introns that are contiguous in a given predicted transcript. When comparing two predictions three differentiated sets are produced: the set of IAs that are identical in both transcripts, and the two sets of IAs that are present in one prediction and not in the other (see Figure 2). If the boundaries of the exons do not agree between two predictions the produced IA contains only the exonic sequence that is common to both predictions, i.e., an intersecting IA contains the sequence and exon-structure on which both predictors agree (see Figure 2). An intron assembly represents naturally the entities to be tested by RT-PCR, where an exon-pair, separated by one or more introns and by not more than about 1 Kb, is tested for amplification in cDNA tissue libraries. As each IA contains the maximal set of introns in a given genomic locus that fall in one of the different categories of the Venn diagram, we picked up one exon-pair from each IA, making sure to test different genes in each Venn diagram.

Combining the operations of set-difference 'not', set-intersection 'and', and set-union '+', we populated the Venn diagram. For instance: the subset 'E not (T+S)' was generated by first obtaining the union of T and S predictions (T+S) and then comparing the Ensembl predictions with this latter set, hence 'E not (T+S)' contains Ensembl IAs that are not in TWINSCAN or SGP2. Similarly, we obtained the subset '(S and T) not E' by first calculating the intersection of S and T, and then calculating the set-difference against E. Note that the set-difference is non-commutative, as we keep elements from one set and use the other for comparison, hence S not T ? T not S. These operations divide the subsets into three types: (1) orphan subsets, formed by those elements that are in one set but not in any of the other two, (2) the two-way intersection subsets, formed by those elements that are in two sets and not in the third one, and (3) the triple intersection, formed by those elements that are in the three sets. Figure 1B shows the number of IAs in each of the subsets (see also Table 1).

When considering the Ensembl predictions we introduced a slight modification of the operations, as one Ensembl gene may have more than one transcript (see Figure 2b). We defined the intersecting IAs as the longest non-redundant common IA between the transcripts from either prediction. That is, from all the redundant intron assemblies in an Ensembl gene that are also in a prediction from SGP2 or TWINSCAN, we took the longest one. By the concept of redundancy of two intron assemblies we mean that they have the same splicing structure or one is included in the structure of the other, allowing for mismatches in the exon edges. Likewise, for the set difference involving Ensembl predictions we took the longest IA from all the ones in an Ensembl gene that were novel with respect to the *de novo *prediction. The inverse case works similarly: an IA in TWINSCAN or SGP2 which is not in an Ensembl gene is the longest IA that is novel with respect to all the Ensembl transcripts in that gene.

We classified IAs according to their position relative to the predictions from the other set against which they are compared. We considered an IA to be:

• Intergenic: if it falls outside the genomic extension of any of the predictions from the other set.

• Bridge: if it bridges between two different predictions in the other set.

• Intronic: if it extends one or more introns of the excluded prediction, i.e., the exons of the IA fall in the introns of the other prediction.

• External: if it extends the 5' or 3' of the other prediction.

**Figure 4:**
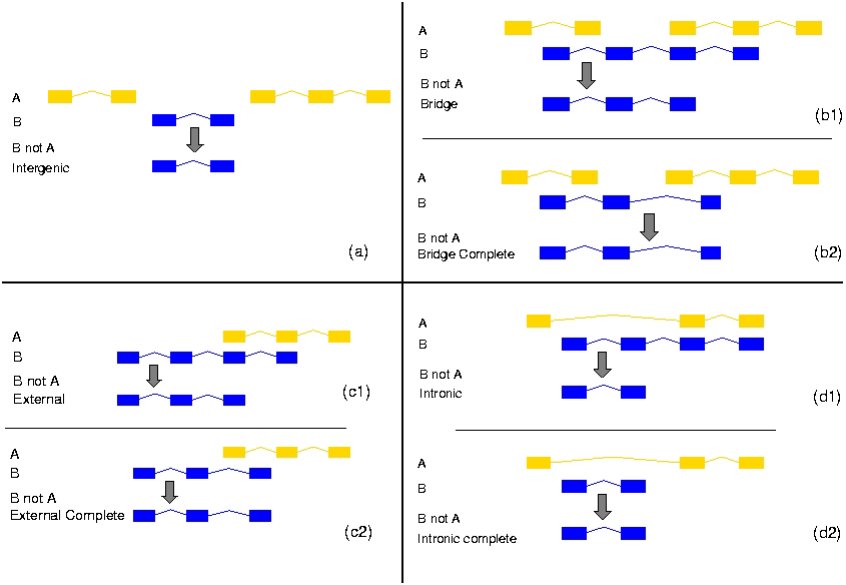
**Classification of intron assemblies. **We classified the intron assemblies that were novel with respect to a reference set according to their position relative to the other set against which we do the comparison. A novel IA can (a) fall between the genomic extent of two predictions (intergenic), (b1) bridge across two predictions (bridge), (c1) overlap the 5' or the 3' end of one prediction (external), and (d1) fall within one or more introns of another prediction (intronic). Additionally, novel IAs are labelled as complete when they are a complete ATG-to-STOP prediction: (b2), (c2) and (d2).

In Table S1 we give the distribution of the IAs according to their relative position (see Figure 4). We observe that while SGP2 produces approximately the same proportion of external and intronic orphan IAs, TWINSCAN produces many more intronic ones.

Finally, IAs were further classified according to whether they are complete, i.e., they represent a complete ATG-to-STOP prediction in at least one set (Figure 4). Table S2 presents the number of complete IAs from each set. TWINSCAN predictions produced more complete novel genes within introns than SGP2, whereas SGP2 predicts more complete novel genes that extend the genomic span of other genes. Furthermore, more than 70% of the complete intergenic orphan IAs from all programs have either two or three exons. Finally, analysing the triple intersection of the prediction sets we find 10,650 IAs that are common to the three sets from which 8,837 (83%) are complete. In contrast to the complete IAs predicted by only one program, 55% of those predicted by all programs have more than 3 exons.

### Comparison to chicken expression data

We compared the prediction sets against 13,880 chicken cDNAs annotated as coding for protein and 4,154 cDNAs annotated as non-coding [12,25]. We used Exonerate in ungapped mode to directly compare the nucleotide sequence of the gene predictions against the cDNA set. We considered a cDNA to be 'found' if there was an alignment with more than 90% identity over more than 60 bp. The results showed that Ensembl and SGP2 included 79% and TWINSCAN included 77% of the coding cDNAs, whereas they included a 17%, 20% and 30% of non-coding cDNAs, respectively. Additionally, we found that 8,126 (28%) SGP2 genes, 7,037 (40%) Ensembl genes and 7,728 (27%) TWINSCAN genes aligned against the coding cDNAs; whereas 838 (3%) SGP2 genes, 1,186 (7%) Ensembl genes and 722 (2.5%) TWINSCAN genes aligned against the cDNAs labelled as non-coding. The three prediction sets together found a total of 12,009 (86.5%) of coding cDNAs, with a common set of 9,597 (69%) coding cDNAs found by all three.

### Primer design

Primers used in the PCR reactions were designed using primer3 and filtered using ePCR. For each IA in each tested set, two exons were selected for primer placement. This selection was made ensuring that the exons selected for primer placement were of adequate length to generate likely primers, and that there was a sufficient amount of coding sequence to give an amplicon length in the desired range. Additional checks were performed in cases where one prediction method included a coding exon not suggested by the other two prediction methods in the same transcript. In those cases, the length of the tentative exons was accounted for by what exons were selected for primer placement, helping reduce failure due to primers being placed too far apart in the actual transcript to get successful amplification. All designed primers and primer pairs were filtered for mispriming using the entire chicken genome to find potential priming locations. Primers returning priming locations not overlapping coordinates of the target gene were rejected. From all passing primer pairs for a given IA, the pair giving the longest expected product in the requested range was selected for amplification. The selection of targets appearing on each plate was done completely randomly among all targets returning at least one designed primer pair passing all criteria. The primer selection parameters used for the design of Plate 1 were consistent with primer3 defaults, except: Tm range was set to (63, 65), product length range was set to (300, 600), and primer length range was set to (23, 28) with 24 being optimal. The primer selection parameters used for the design of Plates 2–5 were also consistent with primer3 defaults, except: GC content range was set to (30, 70) with 50 being optimal, Tm range was set to (59, 62), product length range was set to (150, 500), and primer length range was set to (17, 27) with 20 being optimal.

## Experimental verification of predictions by RT-PCR

### cDNA preparation

Multiple organs (brain, liver, heart, spleen, lung, kidney, muscle, tongue, trachea, crop, proventriculus, gizzard, gall bladder, small-intestine, pancreas, caeca, mesentery, ovary, oviduct and testis) of an adult male and two egg-laying females of the "Bleue de Hollande" strain were collected soon after sacrifice. Total RNA was prepared from frozen tissues using TRIzol Reagent (Invitrogen) according to manufacturers' instructions. The quality of all RNA samples was checked using an Agilent 2100 Bioanalyzer (Agilent Technologies) and by PCR using pairs of oligos designed in four CNGs (Conserved Non-Genic Sequences) conserved between GGA1 and HSA21 [29,30], as indicators of possible genomic DNA contamination. Total RNA was converted to cDNA using Superscript II (Invitrogen) primed with random primers. For each tissue in the study, 5 μg of total RNA was converted to cDNA.

### Experimental verification

Predictions of chicken genes were assayed experimentally by RT-PCR as previously described and modified [9,16,31]. Similar amounts of 12 *Gallus gallus *cDNAs (brain, liver, heart, spleen, lung, kidney, muscle, proventriculus, small intestine, caeca, ovary and testis, final dilution 1000x) were mixed with JumpStart REDTaq ReadyMix (Sigma) and 4 ng/ul primers (Sigma-Genosys) with a BioMek 2000 robot (Beckman). The ten first cycles of PCR amplification were performed with a touchdown annealing temperatures decreasing from 60 to 50°C; annealing temperature of the next 30 cycles was carried out at 50°C. Amplimers were separated on "Ready to Run" precast gels (Pharmacia) and sequenced. This procedure was used to experimentally assay 456 exon-exon junctions of chicken predictions. The later are representative of each subsets of predictions found in the Venn diagram of the three sets of predictions studied, i.e., the Ensembl, TWINSCAN and SGP2 set (see Table S3, and Table S4 for the transcripts used as internal controls). The sequences of the amplified exon-exon junctions can be obtained from the web site .

## Authors' contributions

EE with help from AR and MRB led the writing and preparation of the manuscript. RC, EB, SEA, PF, DDS and RG contributed to the text and overall layout. MRB conceived the Venn diagram approach for the experiments in chicken and chose the target sets, with input from DDS and EE. EE generated the chicken intron assemblies and analysed the experimental results. RC, PF, EE, GP, FC generated the gene predictions and performed several analyses on them. The generation of primers was carried out by DDS, MRB and EB for chicken. AR and CW collected the tissues and prepared the RNAs/cDNAs. EJH, JMB, CW carried out the RT-PCR experiments with supervision from AR, JR, EB and SEA. The analysis of the chicken predictions was coordinated by RG, MRB and EB.

## Supplementary Material

Additional File 1 Supplementary information: distribution of intron-assemblies according to relative positions; details on the RT-PCR test and control sets; accuracy evaluation of the human-based and tetraodon-based chicken gene predictions. Click here for file

## Figures and Tables

**Table 1:** distribution of intron assemblies (IAs) for each of the 7 subsets of the Venn diagram of the three prediction sets: Ensembl (E), SGP2 (S) and TWINSCAN (T) (see also Figure 1). The number of transcripts from each prediction set participating in the intron assemblies is indicated on the right.

**Distribution of intron assemblies**				
Subsets	Number of IAs	Transcripts involved		
		E	S	T
E and S and T	10650	10282	8974	9888
(S and T) not E	4769	0	3930	3924
(E and S) not T	4757	3636	3273	0
(T and E) not S	1748	1592	0	1507
S not (E+T)	25119	0	20740	0
T not (S+E)	27592	0	0	22239
E not (T+S)	13514	11014	0	0

**Table 2:** **Experimentally verified** of intron assemblies (see also Figure 1)

**Experimentally verified intron assemblies**				
	Total tested	No amplimer	Amplimer correctly predicted	Amplimer but junction not correctly predicted
S and T and E	20	3 (15%)	16 (80%)	1 (5%)
(S and T) not E	76	40 (53%)	27 (35%)	9 (12%)
(E and S) not T	64	12 (19%)	44 (69%)	8 (12%)
(T and E) not S	40	14 (35%)	22 (55%)	4 (10%)
S not (T + E)	88	67 (76%)	6 (7%)	15 (17%)
T not (S + E)	96	83 (86%)	9 (9%)	4 (5%)
E not (T + S)	30	7 (23.3%)	16 (53.3%)	7 (23.3%)

**Table 3:** Experimental verification of IAs corresponding to Ensembl 5' extensions. The extensions are separated according to whether the 5'-most Ensembl exon also existed in TWINSCAN and/or SGP2 (linked) or not (unlinked) (see Figure 3).

**Experimentally verified Ensembl extensions**				
Ensembl extensions	Total tested	No amplimer	Amplimer correctly predicted	Amplimer but junction not correctly predicted
linked	60	36 (60%)	11 (18%)	13(22%)
unlinked	29	27 (93%)	2 (7%)	0
