# Supplementary material for: Gene finding in the chicken genome
Source: BMC Bioinformatics. 2005 May 30;6:131. doi: 10.1186/1471-2105-6-131 (PMC1174864; doi:10.1186/1471-2105-6-131)
Supplement: Additional File 1 — Supplementary information: distribution of intron-assemblies according to relative positions; details on the RT-PCR test and control sets; accuracy evaluation of the human-based and tetraodon-based chicken gene predictions. [file 1471-2105-6-131-S1.doc]

**Supplementary material for**

**'Gene finding in the chicken genome'**

Eduardo Eyras^1^, Alexandre Reymond^2,3^, Robert Castelo^1^, Jacqueline M. Bye^4^, Francisco Camara^1^, Paul Flicek^5^, Elizabeth J. Huckle^4^ , Genis Parra^1^, David D. Shteynberg^5^, Carine Wyss^2^, Jane Rogers^4^, Stylianos E. Antonarakis^2^, Ewan Birney^6^, Roderic Guigo^1^ and Michael R. Brent^5^.

^1^*Research Group in Biomedical Informatics, Institut Municipal d'Investigacio Medica/Universitat Pompeu Fabra/Centre de Regulacio Genomica, E08003 Barcelona, Catalonia, Spain.*

^2^*Department of Genetic Medicine and Development, University of Geneva, Medical School and University Hospital of Geneva, CMU, 1, rue Michel Servet, 1211 Geneva, Switzerland.*

^3^*Present address: Center for Integrative Genomics, University of Lausanne, 1015 Lausanne, Switzerland.*

^4^*The Wellcome Trust Sanger Institute, Wellcome Trust Genome Campus, Hinxton, Cambridge CB10 1SA, UK.*

^5^*Laboratory for Computational Genomics, Campus Box 1045, Washington University, One Brookings Drive, St Louis, Missouri 63130, USA.*

^6^*EBI, Wellcome Trust Genome Campus, Hinxton, Cambridge CB10 1SD, UK.*

**Supplementary tables**

|  | Intergenic | Bridge | External | Intronic |
| --- | --- | --- | --- | --- |
| S not (E+T) | 6327 (25%) | 169(1%) | 8688 (35%) | 9935 (39%) |
| T not (S+E) | 5976 (22%) | 130 | 5576 (20%) | 15910 (58%) |
| E not (T+S) | 793 (6%) | 29 | 1286 (10%) | 11406 (84%) |
| (S and T) not E | 1711 (36%) | 3 | 1665 (35%) | 1390 (29%) |
| (E and S) not T | 403 (8%) | 13 | 1024 (21%) | 3317 (70%) |
| (T and E) not S | 54 (3%) | 4 | 231 (13%) | 1459 (83%) |

Table S1: Distribution of intron assemblies (IAs) according to their position relative to the other predictions. We give the number of 2-way intersection IAs and IAs unique to one set. Each entry also shows the number of IAs as a percentage of the total IAs in the same prediction set (i.e. the row total).

| Complete IAs | Total | Intergenic | Bridge | External | Intronic |
| --- | --- | --- | --- | --- | --- |
| S not (E+T) | 10607 | 6327 | 53 | 2844 | 1383 |
| T not (S+E) | 11560 | 5976 | 45 | 1776 | 3763 |
| E not (T+S) | 1862 | 793 | 3 | 311 | 755 |
| (S and T) not E | 2474 | 1711 | 1 | 371 | 391 |
| (E and S) not T | 1122 | 403 | 13 | 214 | 492 |
| (T and E) not S | 432 | 54 | 0 | 53 | 325 |

Table S2: Distribution of complete intron assemblies (IAs). Complete IAs are complete gene structures, i.e., ATG-to-stop, missed by the other programs.

| Plate | Tested set |
| --- | --- |
| 1 | 96 randomly picked Ensembl genes |
| 2 | 90 complete intergenic IAs ( 46 (S and E) not T, 34 (E and S) not T, 10 (T and E) not S ) + 6 controls |
| 3 | Ensembl extensions ( 60 linked + 30 unlinked) + 6 controls |
| 4 | 30 randomly picked IAs from each 2-way intersection + 6 controls |
| 5 | 30 randomly picked IAs from each orphan set + 6 controls |
| Extra plate 1 | 20 triple intersections, 66 extra TWINSCAN orphan IAs and 10 extra SGP2 orphan IAs |
| Extra plate 2 | 48 extra SGP2 orphan IAs |

Table S3: Five plus two plates with primers from chicken predictions were tested. The first plate containing Ensembl random predictions was tested for the analysis of the chicken genome, whereas the other plates were specifically developed for this project. We give for each Plate the number and types of predictions tested.

| Ensembl transcript | Ensembl gene | cDNA | Protein | Gene name/description |
| --- | --- | --- | --- | --- |
| ENSGALT00000008459 | ENSGALG00000005272 | NM_204227 | Q8AYD0 | Cadherin |
| ENSGALT00000010649 | ENSGALG00000006598 |  | Q98930 | SORL |
| ENSGALT00000012893 | ENSGALG00000007936 | NM_204985 | P51903 | Phosphoglycerate Kinase |
| ENSGALT00000017448 | ENSGALG00000010721 |  | Q98T95 | Zinc finger protein |
| ENSGALT00000023692 | ENSGALG00000014698 | U64963 | Q98943 | CASPASE-2 precursor |
| ENSGALT00000025248 | ENSGALG00000015661 | NM_204654 | Q98ST5 | CocoaCrisp ortholog |

Table S4: The 6 Ensembl transcripts used as internal controls in the RT-PCR experiments.

| IAs derived from Ensembl random set | Total | Negatives | Positives |
| --- | --- | --- | --- |
| E not (T + S) | 5 | 3(60%) | 2(40%) |
| (E and S) not T | 3 | 0 | 3 (100%) |
| (T and E) not S | 0 |  |  |
| E and S and T | 12 | 0 | 12 (100%) |

Table S5: In this table we split the results of the tested exon-exon junctions from the set or randomly picked Ensembl predictions according to whether they belonged to one of the subsets of the Venn diagram.

| predictor | SNn | SPn | CCn | SNe | SPe | SNSPe | ME | WE | SNg | SPg | SNSPg | MG | WG | JG | SG |
| --- | --- | --- | --- | --- | --- | --- | --- | --- | --- | --- | --- | --- | --- | --- | --- |
| Genscan | 0.89 | 0.72 | 0.79 | 0.71 | 0.6 | 0.65 | 0.15 | 0.29 | 0.08 | 0.05 | 0.07 | 0.02 | 0.22 | 1 | 1.1 |
| Geneid | 0.83 | 0.85 | 0.83 | 0.66 | 0.73 | 0.69 | 0.22 | 0.15 | 0.13 | 0.11 | 0.12 | 0.02 | 0.15 | 1 | 1.06 |
| TWINSCAN | 0.9 | 0.84 | 0.87 | 0.78 | 0.75 | 0.77 | 0.12 | 0.15 | 0.2 | 0.15 | 0.18 | 0.02 | 0.12 | 1 | 1.17 |
| TWINSCAN* | 0.9 | 0.85 | 0.87 | 0.78 | 0.76 | 0.77 | 0.12 | 0.15 | 0.23 | 0.18 | 0.2 | 0.02 | 0.1 | 1 | 1.18 |
| SGP2 | 0.91 | 0.89 | 0.9 | 0.79 | 0.76 | 0.78 | 0.1 | 0.14 | 0.21 | 0.16 | 0.19 | 0.02 | 0.16 | 1 | 1.13 |
| Ensembl | 0.98 | 0.99 | 0.99 | 0.92 | 0.94 | 0.93 | 0.03 | 0.01 | 0.65 | 0.65 | 0.65 | 0 | 0 | 1 | 1 |

Table S6: Accuracy evaluation of five chicken gene prediction sets. We give the standard gene evaluation parameters of the three predictions studied in this article in chicken together with the results for Genscan and Geneid. The evaluation is given in terms of sensitivity (SN) and specificity (SP) at the nucleotide (SNn and Spn), exon (SNe and SPe) and gene (SNg and SPg) levels, and in terms of a correlation coefficient (CCn), and the average over SN and SP: SNSPe and SNSPg. The table includes the ratios of missing exons (ME) and wrong exons (WE), and the ratios of missing genes (MG) and wrong genes (WG). The last two columns give a measure of the joint genes (JG) and split genes (SG). The evaluation was performed against 525 chicken RefSeq sequences aligned to the chicken genome. For all the programs the predictions were generated on full chromosome sequences and not on individual sequences. To do the evaluation with the Ensembl predictions we took the best predicted transcript, i.e., with highest CCn, for each gene. For our analysis we used the TWINSCAN set. The predictions TWINSCAN* were generated using a corrected intron length model. This latter set shows an improvement in the specificity at the nucleotide, exon and gene levels with respect to TWINSCAN set. Note that TWINSCAN and Ensembl used directly sequence information, and SGP2 used exon-structure information, from the 525 RefSeqs used for this test, hence the table is only an approximation for the evaluation of these three sets.

| Predictor | Informant Genome | SNn | SPn | CCn | SNe | SPe | SNSPe | ME | WE | MG | WG |
| --- | --- | --- | --- | --- | --- | --- | --- | --- | --- | --- | --- |
| SGP2 | human | 0.87 | 0.65 | 0.75 | 0.65 | 0.44 | 0.55 | 0.13 | 0.42 | 0.12 | 0.49 |
| TWINSCAN | human | 0.79 | 0.54 | 0.65 | 0.58 | 0.44 | 0.51 | 0.22 | 0.40 | 0.16 | 0.45 |
| SGP2 | tetraodon | 0.77 | 0.70 | 0.74 | 0.57 | 0.47 | 0.52 | 0.23 | 0.37 | 0.14 | 0.34 |
| TWINSCAN | tetraodon | 0.77 | 0.49 | 0.61 | 0.53 | 0.39 | 0.46 | 0.26 | 0.47 | 0.16 | 0.49 |

Table S7: Comparison of the accuracy of the SGP2 and TWINSCAN gene predictions using Homo sapiens and Tetraodon nigoviridis as informant genomes. The evaluation has been performed against the Ensembl gene set in all G. gallus chromosomes, excluding random chromosomes. See Table S6 for the description of the different accuracy measures.
